# Women’s groups and COVID-19: An evidence review on savings groups in Africa

**DOI:** 10.12688/gatesopenres.13550.1

**Published:** 2022-04-12

**Authors:** Olayinka Adegbite, Leigh Anderson, Sybil Chidiac, Osasuyi Dirisu, Jenna Grzeslo, Julia Hakspiel, Chinmaya Holla, Emily Janoch, Krishna Jafa, Shubha Jayaram, Grace Majara, Tabitha Mulyampiti, Eve Namisango, Eva Noble, Bukola Onyishi, David Panetta, Garima Siwach, Munshi Sulaiman, Rebecca Walcott, Sapna Desai, Thomas de Hoop

**Affiliations:** 1 Independent Consultant, Ibidan, Nigeria; 2 EPAR Evans School of Public Policy and Governance, Seattle, USA; 3 Bill & Melinda Gates Foundation, Seattle, USA; 4 Population Council, Abuja, Nigeria; 5 BRAC USA, New York, USA; 6 MarketShare Associates, Washington DC, USA; 7 International Development Division, American Institutes for Research, Bangalore, India; 8 CARE International, Washington DC, USA; 9 Global Center for Gender Equality, Stanford University, Stanford, USA; 10 CARE International, Kampala, Uganda; 11 School of Women and Gender Studies, Makerere University, Kampala, Uganda; 12 Africa Centre for Systematic Reviews, Makerere University, Kampala, Uganda; 13 Women for Women International, Kampala, Uganda; 14 Women for Women International, Abuja, Nigeria; 15 SEEP Network, Washington DC, USA; 16 International Development Division, American Institutes for Research, Austin, USA; 17 BRAC Uganda, Kampala, Uganda; 18 Population Council, New Delhi, India; 19 International Development Division, American Institutes for Research, Washington DC, USA

**Keywords:** Gender Equality, Women's Groups, Savings Groups, Africa, Nigeria, Uganda, COVID-19, Resilience; Gender; Women’s Empowerment

## Abstract

The coronavirus disease 2019 (COVID-19) pandemic and some of the associated policy responses have resulted in significant gendered impacts that may reverse recent progress in gender equality, including in sub-Saharan Africa. This paper presents emerging evidence from studies in diverse contexts in sub-Saharan Africa —with a deep dive into Nigeria and Uganda—on how COVID-19 has affected women’s groups, especially savings groups, and how these groups have helped mitigate the gendered effects of the pandemic’s and the associated policy responses’ consequences up until April 2021. The synthesis presents evidence that savings groups found ways to continue operating, provided leadership opportunities for women during the pandemic, and mitigated some of the negative economic consequences of COVID-19 on individual savings group members. Savings, credit, and group support from other members all likely contributed to the ability of groups to positively affect the resilience of women’s group member during COVID-19. Households with a female member in a savings group in Nigeria and Uganda have coped with the crisis better than those not in savings groups. While savings groups have shown the potential for resilience during the pandemic, they often faced financial challenges because of decreased savings, which sometimes resulted in the depletion of group assets. Savings groups also contributed to community responses and provided women a platform for leadership. These findings are consistent with a recent evidence synthesis on how past covariate shocks affected women’s groups and their members. We conclude the paper by presenting various policy recommendations to enable savings groups to achieve improvements in women’s empowerment and economic outcomes, and research recommendations to address some of the current evidence gaps on how COVID-19 is affecting women’s groups and their members.

## Disclaimer

The views expressed in this article are those of the authors. Publication in Gates Open Access does not imply endorsement by the Gates Foundation

## Introduction

The coronavirus disease 2019 (COVID-19) pandemic and some of the associated policy responses have resulted in significant gendered impacts that may reverse recent progress in gender equality, including in sub-Saharan Africa. Although mortality rates from COVID-19 are higher for men, women and girls experience more adverse consequences along other health, economic, social, and educational dimensions, with potentially long-term effects ( Burki, 2020; Buvinic *et al.*, 2020; O’Donnell *et al.*, 2021; Wenham *et al.*, 2020). For example, women-owned firms and female workers are disproportionately concentrated in informal sectors, which may lack basic social protections in times of economic crisis, as well as in the hospitality and tourism sectors, which were hit hard by the lockdown ( Copley *et al.*, 2020; O’Donnell *et al.*, 2021; Rochelle Parry & Gordon, 2020). Women are also heavily concentrated in high-risk occupations such as the frontline health care workforce ( Wenham *et al.*, 2020). In addition, various studies suggest that COVID-19 lockdowns may have contributed to increases in gender-based violence ( Agene & Onyishi, 2020; Agüero, 2020; O’Donnell *et al.*, 2021; Peterman *et al.*, 2020) and in women’s burden of unpaid care ( Wenham *et al.*, 2020).

A recent evidence synthesis by the Evidence Consortium on Women’s Groups (ECWG) showed that covariate shocks generally tend to disrupt women’s group activities and reduce group resources, but that women’s groups can support the resilience of group members ( Walcott *et al.*, 2021). While this improved resilience may come at the expense of group resources, the same review suggested that group-based programs may also contribute to mitigating some of the negative economic consequences of COVID-19, especially for women. The findings of the synthesis are consistent with early studies related to the short-term effects of COVID-19 on women’s groups and their members ( Walcott *et al.*, 2021), some of which are included in this review, focused on savings and other women’s groups in sub-Saharan Africa.

In this review, we synthesized emerging evidence from studies in diverse African contexts—with a deep dive into Nigeria and Uganda—on how COVID-19 has affected women’s groups and how these groups have helped mitigate the pandemic’s and the associated policy responses’ consequences in sub-Saharan Africa. ^1^ Women’s groups is an umbrella term commonly used to refer to different models of economic, health, and community groups with a primarily female membership ( Anderson *et al.*, 2019). This review focuses primarily but not exclusively on savings groups; a specific type of women’s group in sub-Saharan Africa. Members of savings groups commonly pool small weekly savings into a common fund, which members can then borrow against, enabling these women to generate social and financial capital ( Namisango *et al.*, 2021). Systematic reviews and impact evaluations show mixed, but promising results of savings groups in improving economic and social outcomes as well as women’s empowerment in sub-Saharan Africa ( Blattmann *et al.*, 2016; de Hoop *et al.*, Forthcoming; Karlan *et al.*, 2017; Steinert *et al.*, 2018, among others).

COVID-19 creates new challenges and opportunities that change the mechanisms through which savings and other women’s groups can achieve impacts. A previous rapid review of the evidence identified three ways in which COVID-19 may affect the functioning and effectiveness of groups, in addition to generating important adaptations in their response to COVID-19 ( de Hoop *et al.*, 2020):

1.    Economic shocks can limit livelihood opportunities for groups and their members, which may result in group dissolution caused by a lack of financial capital. Groups may, however, also provide insurance and business capital through existing savings as well as social protection, which could increase the resilience of women’s group members.

2.    Social distancing requires groups to change their functioning, for example, by replacing physical meetings with virtual meetings, meeting in smaller numbers, or using different means of communication and/or digital payment technologies.

3.    Governments and Non-Governmental Organizations (NGOs) can provide social safety nets and partnerships with groups to manufacture protective equipment, which may increase income opportunities for group members. Women in savings groups may also provide an important voice in guiding local responses to COVID-19 and informing the strategies and actions of governments and NGOs, because the provision of social safety nets and other services through groups could create opportunities for women to build and advance leadership skills ( Janoch, 2020).

This review examines the evidence for these three mechanisms related to the functioning of savings groups and resilience of savings group members after COVID-19, based on data up until April 2021 from a review by Namisango *et al.* (2021) and analyses of monitoring and evaluation and survey data we obtained from Women for Women International (WfWI) in Nigeria ( Siwach *et al.*, 2021). We use a broad definition of evidence, relying on a combination of analyses of longitudinal data from phone-based surveys, descriptive data from international NGOs, and qualitative data on women’s group functioning and resilience. Only very few of the included studies present causal effects using experimental or quasi-experimental designs. Combining the causal and longitudinal analyses from these studies with the mostly descriptive analyses from other studies did, however, enable us to explore in depth how savings and other women’s groups and their members in sub-Saharan Africa were affected by COVID-19 until April 2021.

In this way the paper adds to the literature on how covariate shocks influence women’s groups and their members. This literature points to a tension between programs or strategies that positively affect the resilience of group members (i.e., individuals withstanding the shock) and programs or strategies that positively influence group resilience (i.e., the group functioning coping with the shock) as shown in the evidence synthesis by Walcott *et al.* (2021). Their analysis suggests that women’s groups often are able to contribute to individual resilience through *absorptive capacity* or the ability to cope with or absorb shocks, *adaptive capacity* that includes learning and adaptation to mitigate the effects of shocks, and *transformative capacity* that relates to a systemic adjustment to the status quo that reduces the vulnerability to shocks ( Bene *et al.*, 2015; Tanner *et al.*, 2017; Vaughan & Frankenberger, 2018).

The evidence synthesis provides three main lessons on how groups contributed to individual resilience, while maintaining group resilience:

1.    Savings and other women’s group types likely contributed to mitigating some of the negative economic consequences of COVID-19 on individual savings group members. A longitudinal data analysis indicates that households with a female member in a savings group in Nigeria were statistically significantly less likely to experience food insecurity and more likely to have savings ( Anderson *et al.*, Forthcoming). A different study in Uganda shows statistically significant associations between membership in savings groups, a lower likelihood of suffering income shocks, and a lower likelihood of a reduction in food consumption ( Kansiime *et al.*, 2021). Evidence from a panel data analysis of data from WfWI in Nigeria further suggests that women members of Village Savings and Loan Associations (VSLAs) were more likely to maintain profitable activity after COVID-19. The synthesis also suggested that additional group mentorship to WfWI women’s group members led to a higher likelihood of maintaining profitable activity during COVID-19 ( Siwach *et al.*, 2021). ^2^ However, past members of women’s groups with limited access to recurring financial support, group-based trainings, and associated social networks during the pandemic had a lower likelihood of business continuation during the pandemic than current members ( Siwach *et al.*, 2021).

2.    Women’s savings groups were often able to continue operating during the pandemic, because of digitization, access to mobile money, and by meeting with a smaller number of members. Most savings groups continued meeting after gradual relaxation of lockdowns, but with adaptations for safety and hygiene ( Allen, 2020; CARE, 2020a; CARE, 2020c; Green *et al.*, 2020). However, groups often transitioned to different activities, such as the creation and distribution of personal protective equipment. These and other activities also created new market opportunities to generate income.

3.    Evidence shows that savings groups and other women’s groups have enabled women to obtain leadership positions in the response to COVID-19 ( Agene & Onyishi, 2020; Laouan, 2020). Groups have provided opportunities for women to take on both formal and informal community leadership roles in response to the crisis. They have also served as networks to communicate about how to limit the spread of COVID-19, including the importance of social distancing, hygiene, and safety measures ( Laouan, 2020).

While the analysis indicates that savings groups contributed to individual member resilience in the face of economic challenges related to COVID-19, groups also encountered financial challenges that may threaten their sustainability and effectiveness. These findings suggest that group resilience may require additional investments from policy makers and donors, while recognizing that it will remain important to examine resource constraints and tradeoffs with other investments that aim to achieve similar objectives as savings and other women’s groups. Savings groups likely contributed to the resilience of members after COVID-19, but groups themselves experienced financial challenges because of decreased and/or less regular savings, resulting in the depletion of group assets ( Allen, 2020).

The rest of this paper is structured as follows. We start with a brief background on the initial social protection and lockdown responses, and gradual relaxation measures as COVID-19 evolved in sub-Saharan Africa up until April 2021, and with an emphasis on responses in Nigeria and Uganda. Next, we describe the methods and data sources for the analysis and present the results of our synthesis with an emphasis on resilience, social distancing, and social protection. We finalize the paper with a conclusion that includes various policy and research recommendations related to women’s groups and COVID-19.

## Background

Different responses in Nigeria and Uganda are exemplary of different durations and degrees of strictness in lockdowns, relaxations, and other policy responses to COVID-19 across various African governments. Uganda initially responded to COVID-19 with a lockdown with prolonged closures of schools and universities, a ban on public transportation, a prohibition of travel, the closure of salons, garages, and lodges, and a freeze on private car use until May 2020 ( Anguyo & Storer, 2020). After that, the country gradually reopened different economic sectors and partially reopened schools, colleges, and universities from June 2020, onwards ( “Museveni,” 2020). However, students and teachers in schools were still required to engage in social distancing of two meters or more, wear face masks, and schools were still required to provide access to handwashing stations ( Education Ministry, 2020). ^3^ Nigeria introduced state-level lockdowns in March 2020 with federal travel restrictions and social distancing measures, including a ban on interstate travel and the limiting of market activities to specific days of the week ( Reuters, 2020). After that, the country gradually reopened with “precision” lockdown measures in areas that reported rapid increases in COVID-19 cases in May 2020 ( Reuters, 2020). In a second phase, Nigeria responded with state-led responses, followed by increased gradual relaxation of lockdown such as lifting bans on interstate travel; ending a national curfew; and gradually re-opening schools and places of worship with mandatory mask use, hand-washing stations, and physical distancing between worshippers since June 2020 ( Adepoju, 2020; Andam *et al.*, 2020).

Many countries in sub-Saharan Africa also instituted new or refined existing social protection programs since the onset of the pandemic. These social protection programs include cash transfers and programs focused on food assistance as well as job creation ( Babatunde & Olagunju, 2020; Dixit *et al.*, 2020; Kazeem, 2020; United Nations Development Programme, 2020). For example, Uganda started new social protection interventions in the form of cash transfers and food distribution to cope with lockdowns since April 2020. It expanded jobs under the Urban Cash for Work Program, reaching approximately 500,000 individuals, and distributed agricultural inputs in 124 districts. In Nigeria, the government also distributed cash transfers of $52 to 2.6 million poor and vulnerable households registered with the National Social Register starting in April 2020 ( Babatunde & Olagunju, 2020; Dixit *et al.*, 2020). In addition, it created a special public works program to create temporary jobs for 774,000 Nigerians ( “Federal Govt”, 2020).

We synthesized the evidence on women’s groups and COVID-19 in light of the lockdown, gradual relaxation, and social protection policy responses discussed above. We also present policy and research recommendations that are related to some of the experiences with the policy responses to COVID-19. The goal of the study was, however, not to assess the efficacy of the policies combatting the impact of COVID-19 generally, but rather to assess the effects of the decisions on women’s groups and by extension, women’s group members.

## Methods

We synthesized the findings of various studies on COVID-19 and women’s groups (summarized in Box 1) based on the three mechanisms discussed in the Introduction. We identified studies from three sources: (i) Studies conducted by the ECWG and other grantees of the Bill and Melinda Gates Foundation (BMGF), provided to us by ECWG researchers, ECWG contacts, or Gates Foundation Program Officers, including but not limited to the co-authors of this review; (ii) an external search of studies related to women’s groups and COVID-19; and (iii) outreach to program implementers of women’s group programs in sub-Saharan Africa. The studies conducted by the ECWG and other grantees of BMGF primarily consisted of analyses of phone-based surveys with questions about savings group membership and potential outcomes of savings group membership (e.g., access to credit, savings, food security), analyses of data provided to the ECWG by WfWI, and qualitative research on how savings groups and their members coped with the consequences of COVID-19. The external search of studies used free search terms on Google and Google Scholar that included a) savings groups, b) women’s groups, c) COVID-19, d) financial inclusion, and e) livelihoods. Finally, we reached out to program implementers from existing networks, including but not limited to the co-authors of this review.

**Box 1. Data sources**

▪  An evidence synthesis of groups’ responses to acute covariate shocks and their ability to mitigate the consequences of these shocks before COVID-19 ( Walcott *et al.*, 2021);

▪  Analyses of phone-based surveys on the functioning of savings groups in Malawi, Nigeria, and Uganda during the pandemic ( CARE, 2020a; CARE, 2020b; CARE, 2020c; Crailsheim & Reynolds, 2020);

▪  An analysis of longitudinal in-person (before the pandemic) and four rounds of phone-based LSMS-ISA survey data (after the start of the pandemic) collected in Nigeria [(a more detailed description of the study design, analysis, and detailed results will come out later in 2021 ( Anderson *et al.*, Forthcoming)];

▪  An analysis of one round of phone-based LSMS-ISA survey data [(after the start of the pandemic) collected in Uganda [( World Bank, 2020a)];

▪  An analysis of data from a different phone-based survey in Uganda ( Kansiime *et al.*, 2021);

▪  An analysis of a merged dataset with data from a phone survey with current and past members (program graduates) of WfWI women’s group members to understand how COVID-19 affected their social and economic activities and M&E data with group members’ background characteristics ( Siwach *et al.*, 2021).

▪  An analysis of a merged dataset with the same data from the phone survey with current and past members (program graduates) of WfWI women’s group members and data from a randomized controlled trial to examine the impact of specific components of the women’s group program variation on women’s social and economic outcomes during the pandemic ( Siwach *et al.*, 2021).

▪  A rapid assessment of the effects of COVID-19 on individuals, households, communities, groups, and medium and small enterprises in eight countries in sub-Saharan Africa; the Democratic Republic of Congo, Ghana, Kenya, Malawi, Rwanda, Tanzania, Uganda, and Zambia ( World Vision, 2020);

▪  A survey among refugee and host community savings group members in West Nile, Uganda ( VisionFund, 2020);

▪  A survey among 433 Catholic Relief Services-supported (CRS-supported) savings group representatives in Burkina Faso, Chad, the Gambia, Ghana, Kenya, Madagascar, Mali, Niger, Senegal, Sierra Leone, and Tanzania ( Allen, 2020).

▪  Qualitative data on saving groups functioning and resilience in Mali, Niger, Nigeria, and Uganda ( Agene & Onyishi, 2020; Arasio *et al.*, 2020; Bernardo Andrés & Dawalak, 2020; CARE, 2020b; Laouan, 2020).

Together, this strategy enabled us to identify a considerable number of studies and datasets focused on savings and other women’s groups and COVID-19. These included analyses of cross-sectional phone-based surveys with savings group members and non-members, longitudinal phone-based surveys with data on savings group members and non-members from before and after the pandemic, analyses of datasets that merged M&E data with phone-based surveys and data from randomized controlled trials, as well as phone-based qualitative research studies and surveys on the functioning of savings groups. Box 1 summarizes the studies and data sources we included in the evidence synthesis. 

We started the synthesis of these studies and data sources by examining the validity of the conceptual framework using the findings of a rapid review of the literature on women’s groups and acute covariate shocks that may have had similar (yet less widespread) consequences as COVID-19. This review studied both how these shocks affect women’s groups, as well as their ability to mitigate the effects of shocks for their members and communities ( Walcott *et al.*, 2021).

We combined results from this synthesis with evidence about the functioning of savings groups after COVID-19 using findings from phone-based surveys on how VSLAs and Savings and Internal Lending Communities (SILCs)—prominent forms of savings-led microfinance groups in Africa—and other savings and women’s groups coped with the consequences of COVID-19 across various settings in sub-Saharan Africa ( Allen, 2020; CARE, 2020a; CARE, 2020b; CARE, 2020c; CARE, 2020d; Crailsheim & Reynolds, 2020; World Vision, 2020). We triangulated these findings with qualitative research on how women’s groups coped with the consequences of COVID-19 and the associated lockdown in Mali, Niger, Nigeria, and Uganda ( Agene & Onyishi, 2020; Arasio *et al.*, 2020; Bernardo Andrés & Dawalak, 2020; CARE, 2020a; CARE, 2020b; CARE, 2020d; Laouan, 2020). To examine the economic resilience of women’s group members, we used the results from a data analysis on the association between savings group membership and food security, savings, and credit in Nigeria and Uganda ( Kansiime *et al.*, 2021; World Bank, 2020a), and the association between VSLA membership, mentorship to VSLA members and the likelihood of woman respondents reporting no profitable activity, or being out of business ( Siwach *et al.*, 2021). Finally, we triangulated these findings with the results of 1) analyses to examine correlations between WfWI International women’s group member participant background characteristics and respondents’ self-reported effects of COVID-19, 2) comparisons between the proportion of women reporting negative COVID-19 effects across past and current members of WfWI women’s groups, and 3) analyses to assess the impact of the specific components of the WfWI program variation on women’s social and economic outcomes during the pandemic ( Siwach *et al.*, 2021).

## Results

### Economic shocks and social protection

We found evidence of negative effects of COVID-19 on the resources and activities of women’s groups similar to the results of the evidence synthesis of the effects of past acute covariate shocks ( Walcott *et al.*, 2021); however, most findings up until April 2021 were based on relatively small and likely unrepresentative samples. Most savings groups resumed physical meetings after the initial lockdown, but groups faced challenges related to the depletion of their funds. Many groups disbursed all their funds to help group members and other households in the community meet their immediate needs. Among the groups sampled by CARE in Nigeria, 42 percent used their social funds to support members who needed help, and 15 percent used social funds to purchase hygiene supplies, while 69 percent reported volunteering to help others or participating in groups that organized to protect against COVID-19 ( CARE, 2020d). A survey among CRS-supported savings group representatives in 11 African countries indicated that the support was required not only because of reduced earnings from non-farm livelihoods activities, but also because lockdowns reduced prices for small businesses (because of lower demand) and increased the prices of necessities (because of lower supply [ Allen, 2020]). Savings groups in Karamoja, Uganda also faced major challenges. A rapid assessment of COVID-19’s impacts on these savings groups found that members took loans from their groups to cater to basic needs with the hope of repaying loans when the situation normalizes ( Arasio *et al.*, 2020). This finding is consistent with a survey among 433 CRS-supported savings groups in 11 countries in sub-Saharan Africa, which indicated that 75 percent of savings groups experienced decreased and/or less regular savings ( Allen, 2020). The depletion of group assets in these settings raises concerns about the sustainability of these groups.

Although groups face challenges, access to past savings and credit likely contributed to the resilience of group members during COVID-19. However, group membership was often only able to mitigate a small proportion of COVID-19’s negative economic consequences. Evidence from nationally representative longitudinal panel data based on in-person (before COVID-19) and phone-based (after COVID-19) surveys in Nigeria found that food insecurity in April, June, and August 2020 increased significantly in all survey rounds relative to the in-person General Social Survey (GSS LSMS-ISA) in 2018 (46 percentage points in April 2020; 55 percentage points in June 2020; and 51 percentage points in August 2020 after controlling for various demographic characteristics and savings group membership). The same phone-based surveys indicated that households with a female member in a savings group were less likely to experience food insecurity than households without members in April, June, and August 2020. During this period, households with female savings group members were, on average, 3.7 percentage points less likely ^4^ to report that they faced any food security challenges than non-members after controlling for various baseline and demographic characteristics (from in-person data collected before the start of the phone-based surveys). In August 2020, households with female savings group members were six percentage points less likely to report that they had been unable to eat their preferred food in the last 30 days, 6.7 percentage points less likely to report that they ate less variety of food in the last 30 days, 6.4 percentage points less likely to report that they ran out of food in the last 30 days, 6.3 percentage points less likely to report that they went a whole day without food in the last 30 days, and 4.2 percentage points less likely to report that they had skipped meals in the last 30 days after controlling for various in-person baseline and demographic characteristics.

**Figure 1.**
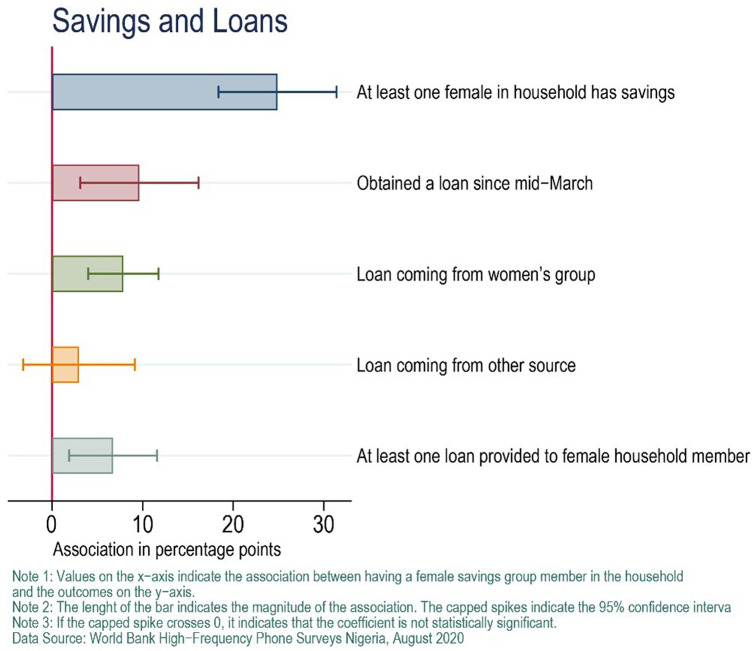
Access to savings and credit in Nigeria after COVID-19.

Access to savings and credit may have contributed to the association between savings group membership and food security in Nigeria. Phone-based surveys showed that households with a female savings group member were 24.5 percentage points more likely to have a female household member with savings in August 2020, 10.8 percentage points more likely to have obtained a loan since March 2020, and 7.6 percentage points more likely to have female household members who had a loan since March 2020. Figure 1 summarizes these findings, which are based on regression models using longitudinal data in which the ECWG controlled for various demographic characteristics ( Anderson *et al.*, Forthcoming).

A study in Uganda suggested membership in savings groups was associated with a lower likelihood of suffering income shocks and a lower likelihood of a reduction in food consumption; however, the findings are likely not representative for Uganda considering the sample was mostly drawn from social media ( Kansiime *et al.*, 2021).

The results are, however, consistent with evidence showing that compulsory savings and flexible credit conditions contribute to the ability of groups to mitigate the negative economic consequences of shocks ( Walcott *et al.*, 2021). Regular savings ensure that members have greater accumulated savings to mitigate the consequences of shocks, while access to information about crop diversification strategies and labor opportunities allowed members to smooth income during shocks ( Demont, 2013; Demont, 2020; Karlan *et al.*, 2017).

However, phone-based surveys from the World Bank in Nigeria and Uganda suggested that only a small percentage of the population obtained credit from their group between March and August 2020. Among the respondents in Nigeria surveyed in August 2020, 19.5% received any credit in August 2020. The loans were predominantly informal, with over 55% of the loans coming from friends or relatives, 9% of the loans coming from banks and microfinance institutions, and 16% from cooperatives and savings groups ( World Bank, 2020b). The World Bank (2020b) reports that these results suggest that Nigerian households may face barriers to obtaining formal loans in the face of a crisis. Among the respondents surveyed in Uganda in August 2020, 22.7% reported receiving any credit, 6.9% reported receiving credit from a VSLA, 4.8 percent reported receiving credit from another savings group, 2.7% reported receiving credit from any other type of formal credit, and 9.8% received credit from a friend or family member ( World Bank, 2020a). Rural households and the poorest were more likely to borrow from savings groups ( World Bank, 2020c). In addition, a large share of the households who borrowed to face the COVID-19 emergency had to borrow because they could not get assistance from family or neighbors ( World Bank, 2020c).

At the same time, most of the CRS-supported savings groups continued to issue loans to their members during COVID-19. Of the 433 savings groups that were included in the survey in 11 African countries, 74% were still issuing loans ( Allen, 2020). In addition, savings groups supported by HOPE extended grace periods and rescheduled loan terms for affected clients ( Green *et al.*, 2020).

Various savings groups have also continued to pursue regular activities during the relaxation of the lockdown. Data from CARE suggests that 83 percent of the groups in Malawi and 70 percent of the groups in the Yobe, Jigawa, and Bauchi states of Nigeria were still saving during the lockdown. Although access to loans was lower, 46 percent of the CARE VSLAs in Nigeria and 50 percent of the CARE VSLAs in Malawi still offered loans ( CARE, 2020b; CARE, 2020c; CARE, 2020d).

Savings group members may be able to rely on previous savings in the short term, but accumulating new savings is likely to be disrupted during COVID-19, though this may differ by population. For example, in Uganda, only 1% of savings group members of a VisionFund program in refugee host communities were able to save the same amount as before the pandemic, compared with 31% of refugee members of savings groups of the same VisionFund program. Although difficult to explain, this may indicate that refugee groups were better able to adapt to newly established meeting guidelines ( Crailsheim & Reynolds, 2020). The same study showed that the average share-out amount of VSLA funds in the West Nile in Uganda increased even at the start of COVID-19 ( Crailsheim & Reynolds, 2020).

Findings from an analysis of WfWI data suggest that women’s group mentorship and a continual support system can provide additional benefits to women’s group members during crises ( Siwach *et al.*, 2021). The data analysis suggests a positive correlation between VSLA membership and economic resilience after COVID-19. However, the data indicate that savings and credit are not the only factor determining resilience. Findings from a sub-sample of women for whom data were collected in an ongoing randomized controlled trial and COVID-19 focused phone-based surveys suggest that group mentorship led to a 20 percentage-point lower likelihood of no profitable activity, but individual mentorship had no statistically significant impact on any resilience-related outcomes ( Siwach *et al.*, 2021). ^5^ The latter finding indicates that a combination of mentorship and group support may be critical to achieve economic resilience during a crisis such as COVID-19.

Only limited in-depth or quantitative research is available on how women’s groups may have contributed to women’s social empowerment. However, qualitative evidence from Ghana, Mozambique, and Uganda indicates that pairing savings groups with child protection interventions may contribute to the effective dissemination of information about child marriage, child labor, the importance of girls’ education, and violence against children during COVID-19 ( World Vision, 2020).

### Social distancing

At the beginning of the pandemic, savings groups supported by CARE in Uganda suspended their meetings and CARE encouraged groups with bank accounts to use mobile money. This experience is consistent with the evidence from past shocks, such as the Ebola epidemic in Liberia, during which the financial activities of groups were disrupted ( Langlay, 2014, p. 30). However, evidence from Uganda also shows variation in group responses across populations: in a VisionFund program in Uganda, 80% of groups in refugee settlements continued to meet in small groups, while only 45% reported the same among the host communities ( Crailsheim & Reynolds, 2020).

Most savings groups supported by CRS and HOPE continued meeting during the pandemic as well. A survey among 433 representatives of savings groups implemented by CRS in 11 African countries indicates that 81% of savings groups continued meeting with adaptations for safety and hygiene, 9% of savings groups stopped meeting without sharing out savings, 6% shared out savings and stopped meetings, and one percent continued meeting without modifications ( Allen, 2020). Of the groups that decided to continue meeting, 47% reported that they considered the risk acceptable, 39% needed to ensure that outstanding loans were repaid, 35% reported that their groups needed to get ready for share-out, and 35% listed other reasons ( Allen, 2020). A survey of 5,000 savings groups implemented by HOPE also indicated that 85% of the groups continued meeting, though many with adaptations to abide by social distancing guidelines ( Green *et al.*, 2020).

In Malawi, Nigeria, and Uganda, savings groups and other women’s groups increased the frequency of meetings after the gradual relaxation of the lockdown. For example, CARE reports that savings groups in Uganda started meeting again with strict adherence to government standard operating procedures. In Nigeria, 39% of CARE-supported savings groups adapted meetings, and in Malawi, the rate was 52% ; however, caution is needed in interpreting these findings because of the small sample size ( CARE, 2020a; CARE, 2020c).

Digitization likely also contributed to savings groups’ ability to continue meeting during the pandemic. For example, CARE-supported urban VSLAs increased their use of mobile money after CARE encouraged VSLAs with bank accounts to use mobile money to save and let members access their savings. Some groups also updated their records via WhatsApp. However, limited evidence exists on the ability of digitized groups to achieve the same results as groups meeting physically, especially as they relate to outcomes such as leadership, solidarity, and women’s social empowerment or productivity and time savings through shared labor ( de Hoop *et al.*, 2020).

Importantly, as the number of digital meetings increases, limited access to technology and other social barriers could result in the exclusion of marginalized women from savings and other women’s groups ( CARE, 2020c; de Hoop *et al.*, 2020). For example, less than 25 percent of women in northern Uganda and less than 40 percent of women in the northeast and northwest zones of Nigeria own mobile phones ( UBOS & ICF, 2018; NPC & ICF, 2019). Findex data from 2014 further suggests that relatively low proportions of women (less than 20%) in Nigeria reported using a mobile phone or the internet to conduct financial transactions or had a mobile money account, compared with more than 50% of women in Uganda.

### Partners in community response

Evidence from West Africa indicates that savings groups and other women’s groups provided an avenue for some women to achieve leadership roles in the response to COVID-19. Women’s savings group members in Mali organized to shift their income-generating activities from businesses like selling food at schools—which were no longer open during COVID-19—to making and selling masks, including selling to actors like the World Food Program. Change agents from WfWI-supported women’s groups also shared recommendations on preventive behaviors such as physical distancing, handwashing, and using face masks in Nigeria ( Agene & Onyishi, 2020). While time constraints related to domestic work and time required for caregiving may continue to limit the participation of women in community decisions, women without formal leadership roles are still often active in the COVID-19 response. For example, CARE-supported savings groups in Benin, Mali, and Niger contributed to community-level decision making in response to COVID-19 ( CARE, 2020e).

Across sub-Saharan Africa, savings groups contributed to collective action by building handwashing stations, creating community action plans to prevent COVID-19, and raising awareness about COVID-19 and its prevention from April 2020 onwards ( CARE, 2020e). For example, CARE provided remote trainings to community leaders and program participants on COVID-19 prevention measures, disease symptoms, and how to access health care services in Nigeria ( CARE, 2020a). Of the CRS-supported savings groups that continued to meet, 68% provided water and soap to their members, 64% required members to wash their hands at the beginning of the meeting, 52% at the end of the meetings, and 49% after handling money ( Allen, 2020). In addition, savings group members in Cote d’Ivoire, Mali, and Niger negotiated with private sector companies to obtain more handwashing supplies to use during savings groups meetings ( Bernardo Andrés & Dawalak, 2020). The evidence is consistent with what is known about the role of women’s groups during the community response to Ebola in the Democratic Republic of Congo (DRC), where the World Health Organization trained women’s group representatives to spread awareness and share information about vaccines, contact tracing, treatment, and the vulnerability of women and children to the disease ( World Health Organization, 2018). However, limited evidence is available on the population reach and effectiveness of information about COVID-19 disseminated through women’s groups.

Savings groups also contributed to COVID-19 response efforts by producing masks and personal protective equipment (PPE) and by introducing modified safety and hygiene procedures. For example, women savings group members in the Democratic Republic of Congo, Mali, and Nigeria are producing masks for the group members as well as the most vulnerable populations in their communities. Producing these masks could help in generating additional income for individuals and the group, considering that group members often sell the masks they produce to obtain revenue and make contributions to group social funds ( Agene & Onyishi, 2020; Laouan, 2020).

Evidence from various settings also indicates that savings group members engaged in collective action to support the community ( Bernardo Andrés & Dawalak, 2020). For example, qualitative findings from Nigeria indicate that women’s groups may have helped to provide support to members in acute need, connecting women with new income opportunities and helping to reduce gender-based violence ( Agene & Onyishi, 2020).

## Discussion

Evidence across sub-Saharan Africa indicates that savings groups have supported the economic resilience of their members during COVID-19. Results from various studies suggest that households with savings group members were less likely to experience food insecurity and had a lower likelihood of suffering income shocks, a reduction in food consumption, and reductions in profitability than households without savings group members ( Anderson *et al.*, Forthcoming; Kansiime *et al.*, 2021; Siwach *et al.*, 2021). Savings and credit likely contributed to improvements in economic resilience for households with savings group members, but evidence from Nigeria indicates that mentorship and continued support from other group members likely contributed to additional economic benefits for women’s group members ( Siwach *et al.*, 2021).

While evidence indicates that savings groups in sub-Saharan Africa continued meeting and have supported household resilience during the COVID-19 crisis, savings groups face challenges and limited resources that threaten their sustainability and effectiveness over time. Due to increased disbursements to members and non-members in need of cash during the crisis, savings group funds have been depleted in some cases. The economic crisis has disrupted the accumulation of new savings because of reduced employment opportunities and income, and, while the use of digital meetings and mobile money has helped those with access, the shift to digitization has created barriers for the most marginalized women ( CARE, 2020c; de Hoop *et al.*, 2020).

Savings groups also contributed to collective action and opportunities for women to obtain leadership roles in response to the pandemic. While we only found limited evidence on the impact of women’s groups on social empowerment during COVID-19, women’s savings group members contributed to collective action by building handwashing stations, creating community action plans to prevent COVID-19, and raising awareness. Women’s leadership roles often went hand in hand with the creation of additional income generation opportunities, for example by producing and selling masks and PPE.

### Policy recommendations

We present various recommendations for policymakers who aim to strengthen savings and other women’s groups with the objective of achieving improvements in women’s empowerment and economic outcomes. These recommendations are consistent with those presented in the brief on savings groups and COVID-19 published previously ( Namisango *et al.*, 2021). While the recommendations suggest the importance for policymakers, NGOs, and donors to review their current efforts to support women’s groups and identify areas where they could adapt or strengthen their engagement, it will remain important to examine resource constraints and tradeoffs with other investments that aim to achieve similar objectives.

***Policy and programmatic implications for governments***

■    In the short term, combining savings groups with cash assistance, voucher assistance, and food aid could help women’s group members and households manage the effects of the crisis, protect member assets, and recapitalize savings groups, which is vital to response and recovery efforts. A dedicated public fund could target savings group members who face sharp reductions in income and fall below a vulnerability threshold due to COVID-19. Governments could determine this vulnerability threshold based on a combination of real-time group financial data (such as savings rates, loan disbursement/repayment behavior, or group liquidity, including data on mobile money); mobility indicators (Google mobility data can be used as a proxy for social distancing and livelihoods opportunities); and disaggregated data on food security. Ensuring low-cost targeting will require the use of savings groups MIS data and real-time data (i.e., mobile money). Regardless of the specific mechanism, which may differ depending on the context, combining savings groups with emergency public assistance will require clear communications and community engagement in addition to effective targeting and the choice of the right transfer mechanism (e.g., cash transfers, vouchers, or asset transfers).

■    In the long term, integrating savings groups into social protection programs may have broad, longstanding benefits because savings groups could strengthen the effectiveness of social protection programs and create interaction effects. Targeting savings groups could build the resilience of groups, their members, and their communities to future emergencies. SEEP (2018) identified 20 social protection programs and policies in sub-Saharan Africa with a savings group component; governments could draw lessons from this study and examine whether further integrating savings groups into national safety nets could meet program goals in a more effective and efficient way, though more evidence is needed to examine the synergies of social protection and savings group programs.

■    Flexibility in external loan repayments for those groups with access to formal credit during COVID-19 will help women and households cope with the current crisis and possibly increase their resilience to future shocks. This flexibility could help women and households with access to formal credit cope with the current crisis and possibly increase their resilience to withstand future shocks.

■    Higher-quality data and more frequent data collection on women’s groups supported by African governments could generate additional evidence to guide decision making on government-supported women’s groups. Current evidence primarily focuses on NGO-supported groups, and the evidence presented in this paper may not necessarily apply to groups supported by governments.

***Policy and programmatic implications for organizations working directly with women’s groups***

■    Prioritizing women’s leadership in the community response to COVID-19 while ensuring social distancing and access to PPE could enable women to obtain leadership positions in the long term. Evidence already shows some examples of how women’s leadership contributes to community-level decision making, which may in turn contribute to women’s empowerment in the long term. 

■    Ensure that past savings group members have access to a continual support system. Graduates from savings group programs could for example maintain their assigned program groups or form new groups to create an accountability structure. Without such support, the resilience of graduates from group programs is likely limited during and after large negative shocks. 

■    Create support systems for women to achieve economic independence through savings groups and group mentorships. Additional life skills training as part of savings groups may also affect women’s self-efficacy, which can contribute to resilience during similar covariate shocks.

■    Pay group members for their services when providing access to vital products, entitlements, and information. This could, for example, happen through public procurement of such services.

***Policy and programmatic implications for donors***

■    Invest in national management information systems (MIS) and the national mapping or registration of groups in partnership with governments to support long-term monitoring and build the capacity of governments to use MIS to guide decision-making. Such systems could help with the tracking of savings group MIS data on savings and other key vulnerability indicators for groups and group members for whom it is currently not feasible to track data. Recent advances in digital savings group applications, in-app and SMS messaging, and remote surveys have driven down data collection costs ( SEEP, 2019) and may allow donors and governments to invest in lower-cost solutions to generate data, including data based on MIS for savings groups. National maps, registries, or databases would also support coordination among stakeholders and enable governments to better support and engage women’s groups in emergency prevention, response, and recovery efforts as well as help group members monitor their group’s performance and needs.

■    Leveraging the learning and insights coming from the SEEP peer learning group on savings groups, women, and COVID-19 could enable donors, governments, and practitioners to guide COVID-19 response and recovery efforts related to savings groups. The SEEP peer learning group will bring together a group of diverse public and private sector stakeholders directly involved in implementing gender-intentional COVID-19 response and recovery efforts through savings groups for a collaborative learning period of about 18 months.

### Research recommendations

This paper presents emerging evidence on savings groups and COVID-19 from studies in diverse African contexts, but major evidence gaps remain. It therefore remains important to continue investing in research on how COVID-19 affects women’s groups and their members if the goal is to fill these gaps. Below we present various recommendations on priorities to consider in this longer-term research on women’s groups and COVID-19.

■    Conduct research on group savings and other vulnerability indicators, and how these link to COVID-19 policy responses, at different stages of the pandemic. Helping governments and NGOs identify savings groups that require cash or other support to continue functioning will require data that tracks collective savings and other vulnerability indicators during and after COVID-19. Analyzing such trends over time and across geographies, for example, by using MIS data on savings group functioning and vulnerability of members, can provide insights into variations across savings group functioning. For example, government agencies could set a vulnerability threshold below which savings group members would receive either flexible credit, cash, or asset transfers (depending on the context) with appropriate monitoring. Such thresholds may also enable researchers to estimate the impact of cash or asset transfers and credit using a regression discontinuity design, which is important to examine how savings and other women’s groups can leverage cash or asset transfers during a shock and how it affects the outcomes of individual members and their community.

■    Conduct research on how COVID-19 has changed women’s group members’ livelihood options in ways that may endure, including the use of digital savings, their role in emergency response efforts, and the role of savings as insurance, for example to guide decisions about livelihood interventions for women’s group members. Women will continue to face economic challenges in the aftermath of COVID-19 and access to markets and other livelihoods options may help to mitigate some of these consequences.

■    Continuing research on long-term resilience would help to capture the long-term effects and roles of savings and other women’s programs and specific components of women’s group programs. In addition, it would contribute to uncovering the underlying mechanisms driving women’s economic and empowerment outcomes and recovery following negative shocks. Additional rounds of data collection related to existing randomized controlled trials and quasi-experimental studies will likely offer the opportunity to determine longer-term effects of savings and other women’s group programs after COVID-19.

■    Conduct experimental and quasi-experimental research to determine the relative effectiveness and cost-effectiveness of virtual and in-person training and other digital delivery channels in generating positive effects on women’s empowerment and economic outcomes, while taking into consideration equity concerns related to access to technology and digital knowledge. Such impact evaluations could guide and prioritize decisions about the scale-up of trainings in the aftermath of COVID-19, along with qualitative research on group implementation processes and their coping mechanisms.

■    Conduct qualitative research on group processes with a view to inform future programming. This can include research on how group members coped with COVID-19 relative to non-members, and how COVID-19 influenced gender dynamics in the household and community.

## Data availability

No data are associated with this article.

## Footnotes

^1^ The review is heavily based on our previous brief with results from an evidence synthesis on women’s groups and COVID-19 ( Namisango *et al.*, 2021). We include all findings from that evidence synthesis and triangulate those findings with new analyses based on longitudinal monitoring and evaluation and survey data the ECWG obtained from Women for Women International in Nigeria. We provide more details on the latter analysis in a detailed brief ( Siwach *et al.*, 2021).

^2^ This analysis was based on data from a sub-sample of women for whom data were collected for a randomized controlled trial of WfWI’s gendered graduation program and a COVID-19 focused data collection. We need to exercise some caution in interpreting this result, however, because of the relatively small sample size. The full forthcoming findings of the randomized controlled trial will provide more conclusive results.

^3^ We recognize that Uganda reintroduced some of the lockdown measures in May 2021, after a large increase in the number of COVID-19 cases in the country. Since then, Uganda has also gradually relaxed some of the COVID-19 restrictions in July 2021 ( Athumani, 2021), though most schools were still closed in September 2021. This paper focuses on the policy responses to COVID-19 up until April 2021.

^4^ This point estimate is an average across the three rounds of phone-based survey data from April, June, and August 2020 that included measures of food security and based on five survey questions that were included in all phone-based surveys ( World Bank, 2020a). One phone-based survey in July 2020 did not include questions about food security. The five survey questions asked about the ability of households to eat their preferred food, the variety of food consumed by households, whether households ran out of food, whether households went a whole day without food, and whether households had skipped meals (all in the last 30 days).

^5^ The sample size for this study is small, however, because of limited overlap between the two datasets, which poses a limitation, as indicated in Siwach *et al.* (2021).
