# Supplementary material for: Women’s groups and COVID-19: An evidence review on savings groups in Africa
Source: Gates Open Res. 2022 Apr 12;6:47. [Version 1] doi: 10.12688/gatesopenres.13550.1 (PMC9513104; doi:10.12688/gatesopenres.13550.1)
Supplement: Peer review history

# Peer review: Reviewer response for version 1

**DOI:** 10.21956/gatesopenres.14816.r32527

**Published:** 2022-09-26

**Authors:** Agnes R. Quisumbing

**Affiliations:** 1 International Food Policy Research Institute, Washington, DC, USA

The article brings together evidence from different sources on how savings groups in Africa may have mediated the impact of the Covid-19 pandemic on women who were members of those groups. Because the impacts are assessed as of April 2021, the evidence is preliminary. However, the authors do a good job of synthesizing the material from various sources while being appropriately cautious about the ability to draw definitive conclusions. Part of the caution arises from the methodology used by the studies included in the review. Researchers working on Covid-19 have, by necessity, had to be opportunistic: adding on survey rounds (often using phone surveys) to existing data collection efforts, and taking advantage of any exogenous variation in the timing of Covid restrictions to be able to identify causal impacts.

 The authors identify three ways through which savings groups may have contributed to individual resilience “while maintaining group resilience” (authors’ words in quotes; I will return to this important point later). First, various studies show that women who were members of savings groups were less likely to experience negative outcomes from the pandemic. The authors do not indicate whether these studies refer to past or current membership or correct for self-selection into the groups. It appears that current membership is important; one of the studies cited (Siwach *et al.*, 2021) mentions that past members of groups with limited access to recurring financial support, group based trainings, and associated social networks during the pandemic had a lower likelihood of business continuation compared to current members. Second, savings groups that continued operating were able to do so because of digitization, use of mobile money, and meeting with a smaller number of members. Some groups also undertook new activities (making of PPE) that created new opportunities for their members. Third, savings groups enabled women to obtain leadership positions in the response to Covid-19.

 I will organize the rest of the discussion according to the three ways identified by the authors. Incidentally, the headings in the paper do not correspond directly to the three ways identified and are a bit confusing.

Mitigating negative economic consequences:

 I think that there is substantial evidence that group membership contributes to individual resilience, but whether groups themselves can maintain resilience is unclear. Is this because of lack of evidence? The authors already point out that groups themselves experienced financial challenges because of decreased and/or less regular savings, resulting in the depletion of group assets (citing Allen, 2020). But is there also a possibility of a conflict of interest, in which members will prioritize their own (and their families’) livelihoods first before contributing to the group? I wonder how many of the studies tracked both current and former members, and also importantly, both groups that “survived” and groups that dissolved. A full analysis of group resilience requires following up all groups, not only those that remained intact during the pandemic.

 The authors might want to examine the extent to which the shift to phone surveys biased comparisons to previous surveys that were carried out in person. For example, comparing food security estimates obtained from an in-person survey to those obtained using phone surveys may need to control for possible effects of the survey mode, even if demographic characteristics and group membership are already controlled for.

Changing modalities of group functioning:

 The heading of “social distancing” is not appropriate for this section, which discusses how groups changed their operational modalities to function during the pandemic. This included temporary suspension of meetings during the lockdown, but, in some cases, more frequent meeting afterwards, and digitization. Although social distancing may have been the impetus for the adjustments in group operations, the adjustments themselves show how groups innovated to meet pandemic restrictions as well as their own objectives.

Creating opportunities for leadership and collective action:

 I also suggest replacing the heading of “Partners in community response” with the above “Creating opportunities…” because the latter is more consistent with the three points made earlier in the paper.

 The paper ends with a discussion of policy and programmatic implications and recommendations for research. Although the policy and program implications make sense, they do not follow directly from the evidence review. The authors could have a section on programmatic implications that derive directly from the review in addition to the current policy recommendations section (or the sources of the recommendations, if not from the evidence review, could be more clearly discussed). One recommendation could be on innovations adopted during the pandemic that could be continued (the authors mention digitization, provided that access issues are addressed).

 A minor point: the recommendations are framed for policymakers who aim to strengthen savings and other women’s groups “with the objective of achieving improvements in women’s empowerment and economic outcomes.” Women’s empowerment is barely discussed in this paper, which focuses mostly on economic outcomes and resilience. Why not rephrase this to “women’s resilience and economic outcomes”?

Additional questions and opportunities for development:

 While the paper makes an important contribution to understanding the role of savings groups in the promoting resilience both in the Covid response and in future disasters, there are some gaps in the paper that could be potential areas for future research and programming. For example, on p. 5, the authors allude to a tension between programs and strategies that affect individual resilience and those that maintain group resilience.  The authors then neglect this potential tension in mentioning the three main lessons on how “groups contributed to individual resilience, while maintaining group resilience,” but seem to assume that group resilience is maintained. It would be good to explore possible conflicts of interest between individual and group objectives. Qualitative studies of those who dropped out (individuals) and groups that dissolved would be as insightful as the studies focusing on those which continued operating.

Does the article adequately reference differing views and opinions?

Yes

Are all factual statements correct, and are statements and arguments made adequately supported by citations?

Yes

Is the Open Letter written in accessible language?

Yes

Where applicable, are recommendations and next steps explained clearly for others to follow?

Yes

Is the rationale for the Open Letter provided in sufficient detail?

Yes

Reviewer Expertise:

I am an economist who has worked extensively on gender and intrahousehold issues in LMICs. More recently, I have worked on developing women's empowerment metrics and using them in impact evaluations, including evaluations of group-based programs.

I confirm that I have read this submission and believe that I have an appropriate level of expertise to confirm that it is of an acceptable scientific standard.

# Peer review: Reviewer response for version 1

**DOI:** 10.21956/gatesopenres.14816.r31938

**Published:** 2022-05-24

**Authors:** Kathleen E. Odell

**Affiliations:** 1 Brennan School of Business, Dominican University, River Forest, IL, USA

This article summarizes preliminary evidence on the relationship between women’s groups, especially savings groups, and the effects of the COVID-19 pandemic. This synthesis paper presents evidence from diverse contexts in sub-Saharan Africa regarding the association between group participation and the gendered impacts of the pandemic. The evidence suggests that group membership is correlated with better outcomes across several measures including individual resilience, women’s leadership, and community responses. The authors clearly document their data sources (see Box 1), and are careful to avoid drawing inferences about causation given the methodology of the studies included in the synthesis. Overall, this is a very useful summary of the existing evidence, which raises several follow-up questions and adds to our understanding of how women’s (savings) groups function and their role in response to crises and shocks. 

 The study authors borrow from de Hoop *et al.* (2020) to identify three pathways for interaction between savings group membership and the effects of COVID-19. First, by providing access to insurance and business capital, groups have the potential to increase the resilience of their members. Second, social distancing measures such as the shift to virtual meetings may fundamentally change the operation of groups. Third, groups may work with governments or NGOs on community responses to the pandemic. Evidence from a variety of sources is then organized based upon these three pathways. 

 The evidence on the first mechanism, where group membership supports individual resilience, is quite clear and convincing. 

 The second mechanism is titled “Social distancing,” (e.g.: the section header on page 9); however, the discussion is mostly related to meeting frequency and digital adaptations. It may increase the clarity here to consider retitling the mechanism to “Meeting schedules and Digitization” or something along those lines. This section also seems to be missing a summary or “so-what” section. It makes the case that groups functioned differently as a result of the pandemic, but does not take the next step to discuss how these adaptations might continue after the pandemic or what the implications of the adaptations may be. If possible, I would encourage the authors to expand the discussion in this section – the shift to digital meetings and mobile money is especially compelling given how this may lead to a corresponding shift in the demographics of group participation.

 The third mechanism, on community response, highlights several instances where groups took action within their communities to produce masks, build handwashing stations, and share best practices. These outcomes are quite interesting. The third paragraph, which begins “Savings groups also contributed…” seems somewhat repetitive and could possibly be eliminated or combined with the first paragraph. 

 The paper concludes with a series of policy recommendations for governments, NGOs, and donors, as well as a call for additional research. The policy recommendations seem sensible but do not necessarily flow directly from the research synthesis. In terms of future research, a missing piece for me is research into the implications of the shift to digital meetings, mobile money, and digital communication (i.e. WhatsApp). 

**Additional questions and opportunities for development:**

 On page 5, the authors mention that the literature on groups and covariate shocks points to a “tension” between programs that affect the resilience of individual group members, and programs that affect the resilience of the groups themselves. This is a very interesting idea and one that I would have liked to better understand; however, in my reading of the paper I did not find any additional discussion of this tension. is it possible to further explore this idea?

 Figure 1 summarizes evidence from a phone-based study in Nigeria. I found the figure difficult to interpret, and I’m not sure it adds value to the paper. If the authors find a separate summary of this study helpful (the key findings are already summarized in the text) then a table with the point estimates and confidence intervals may be more legible than the diagram. If the diagram is retained as it is, then there are two typos in Note 2: “lenght” and “interva”.

Does the article adequately reference differing views and opinions?

Yes

Are all factual statements correct, and are statements and arguments made adequately supported by citations?

Yes

Is the Open Letter written in accessible language?

Yes

Where applicable, are recommendations and next steps explained clearly for others to follow?

Yes

Is the rationale for the Open Letter provided in sufficient detail?

Yes

Reviewer Expertise:

My research focuses on economic development and I have written several reports about the impact of microfinance and savings groups.  I am trained as an economist and have long-term interests in program evaluation, poverty alleviation, and sustainable development.

I confirm that I have read this submission and believe that I have an appropriate level of expertise to confirm that it is of an acceptable scientific standard.
